# Enhancement of Optical and Chemical Resistance Properties with a Novel Yellow Quinophthalone Derivative for Image Sensor Colorants

**DOI:** 10.3390/molecules29051100

**Published:** 2024-02-29

**Authors:** Sunwoo Park, Raveendra Jillella, Hyukmin Kwon, Sangwook Park, Hayoon Lee, Kiho Lee, Jongwook Park

**Affiliations:** Integrated Engineering, Department of Chemical Engineering, Kyung Hee University, Gyeonggi 17104, Republic of Korea; mdrafix@khu.ac.kr (S.P.); raveendrajillella123@gmail.com (R.J.); hm531@khu.ac.kr (H.K.); pswook@khu.ac.kr (S.P.); kssarang1@khu.ac.kr (H.L.); kiholee@khu.ac.kr (K.L.)

**Keywords:** quinophthalone derivative, yellow colorant, color filter, image sensor

## Abstract

A novel quinophthalone derivative, 4,5,6,7-tetrachloro-2-(2-(3-hydroxy-1-oxo-1*H*-cyclopenta[*b*]naphthalen-2-yl)quinolin-4-yl)isoindoline-1,3-dione (TCHCQ), was designed and synthesized as a yellow colorant additive for green color filters in image sensors. The characteristics of the new material were evaluated in terms of optical, thermal, and chemical properties under solution and color filter film conditions. TCHCQ exhibited a significantly enhanced molar extinction coefficient in solution, being 1.21 times higher than that of the commercially used yellow colorant Y138. It also demonstrated excellent thermal stability, with a decomposition temperature (T_d_) exceeding 450 °C. Utilizing the nano-pigmentation process, TCHCQ was used to prepare nano-sized particles with an excellent average size of 35 nm. This enabled the fabrication of a color filter film with outstanding properties. The optical properties of the produced film revealed outstanding yellow colorant transmittance of 0.97% at 435 nm and 91.2% at 530 nm. The color filter film exhibited similar optical and thermal stability to Y138, with an improved chemical stability, as evidenced by a ΔE_ab_ value of 0.52. The newly synthesized TCHCQ is considered a promising candidate for use as a yellow colorant additive in image sensor color filters, demonstrating superior optical, thermal, and chemical stability.

## 1. Introduction

Recent advancements in key technologies such as artificial intelligence (AI), big data, and the Internet of Things (IoT) have rapidly progressed, particularly in camera and display devices associated with these technologies. These advancements are evolving based on visual information, and the importance of color filter technology for image sensors is gradually increasing, driven by the significance of visual information. Color filters for image sensors play an important role in allowing specific wavelengths of incident light to pass through, transforming them into optical images [1,2,3]. Achieving high-quality image generation in future image sensors requires sub-micrometer-scale pixel sizes. In such small-sized pixels, optimized absorption and transmission characteristics are essential for creating high-quality images with small-sized color filters. This requires high absorption efficiency and interference prevention at the optimized wavelength. To address this, the development of new materials with a high molar extinction coefficient for colorants is crucial [4,5,6,7,8]. Furthermore, the study of materials with high thermal and chemical properties for colorants is urgently needed to reliably prepare and use components in small-sized pixels [9,10,11,12]. This becomes important for creating stable components in small-sized pixels. These requirements apply to all three colors—red, green, and blue—and particularly for the challenging green color, which often utilizes yellow colorant as an additive to enhance its optical characteristics [13,14,15]. The fundamental optical characteristics required for this yellow colorant include a low transmittance of 5% or less at 435 nm and a high transmittance of over 90% at 530 nm in the film state [16]. In addition to these requirements, for sub-micrometer-scale pixels, there exists the need for properties satisfying color differences (ΔE_ab_ values) of 3 or less, considering the thermal and chemical conditions required in the manufacturing process. This becomes crucial for ensuring the color accuracy under the processing conditions required for heat resistance and chemical stability in sub-micrometer-scale pixels. From this perspective, the existing yellow colorant, primarily based on the quinophthalone moiety with Y138 as a central material, has been commercially available. However, for future image sensor colorants, there is a need for performance improvement, leading to ongoing research for various yellow colorant developments. Therefore, in this study, a new yellow colorant was designed and synthesized based on the moiety containing 4,5,6,7-tetrachloroisoindoline-1,3-dione and a quinoline functional group. The new yellow colorant was designed by substituting the 4,5,6,7-tetrachloroisoindoline-1,3-dione moiety in the para position with respect to the nitrogen atom in the quinoline moiety, and it was synthesized by removing the chloride atom from the 4,5,6,7-tetrachloro-1*H*-indene-1,3(2H)-dione moiety and introducing a naphthyl group. This structural modification aimed to induce changes in the performance of the image sensor colorant by introducing the naphthyl group while maintaining a similar twist angle within the molecule. The newly synthesized yellow colorant was evaluated for its potential as a yellow color filter material by examining its optical properties, thermal stability, and chemical stability in comparison to Y138 in both solution and color filter film states, obtained through the pigmentation process.

## 2. Results and Discussion

**Scheme 1:**
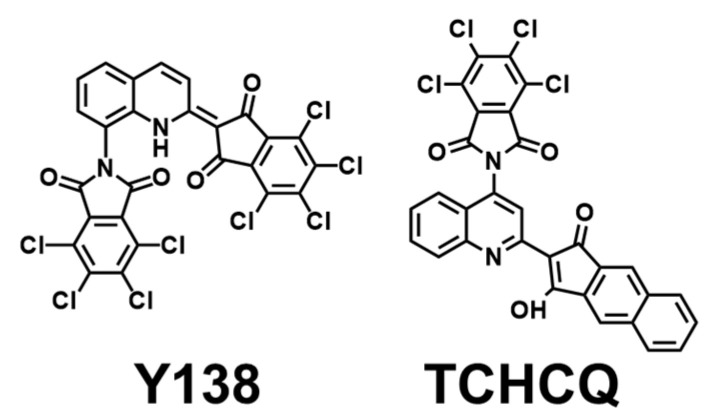
Molecular structures of newly synthesized material and Y138.

**Scheme 2:**
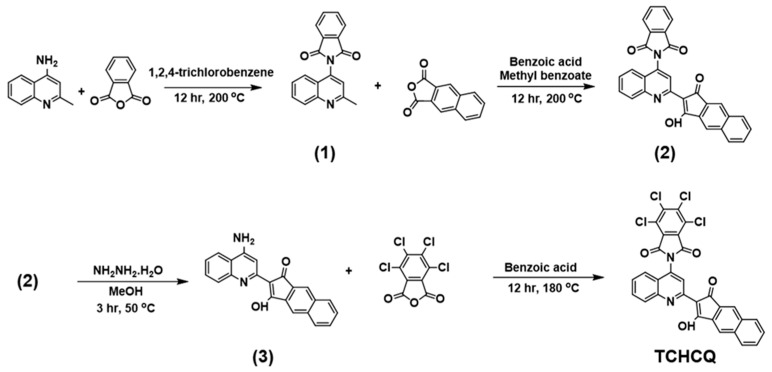
Synthetic routes of newly synthesized compound.

As the pixel size of image sensors decreases, there is a demand for image sensor colorants that can strongly absorb light in specific regions to provide clear images. In this study, we designed a molecular structure based on quinophthalone to develop a new yellow colorant in the field of yellow colorants, compensating for the absorption characteristics of the challenging green colorant among R, G, B colors. Commercial green colorants require high absorption in the 435 nm blue region and high transmittance in the 535 nm region. In particular, the absorption characteristics of the green colorant in the 435 nm blue region are insufficient, and to address this issue, yellow colorants are used in conjunction to compensate for it. The quinophthalone group has been a focus of derivative research due to its relatively high absorption and chemical stability in the 400~500 nm range, with Y138 serving as a representative example. However, even the commercialized Y138 has limitations in its absorption characteristics, especially in the 435 nm blue region. To improve this issue, in this study, we optimized the absorption wavelength range of the organic molecule to induce optimal absorption at 435 nm and enhanced the absorption intensity at 435 nm by adjusting the substitution positions of 4,5,6,7-tetrachloroisoindoline-1,3-dione and the number of chlorine atoms based on Y138. This approach strengthens the interaction with solvent molecules and aids in improving solubility. Additionally, the introduction of a naphthyl group increases the molecular weight, improving thermal stability and enabling absorption in the blue region by increasing the molecular conjugation length. The structure and synthetic route of the newly proposed quinophthalone derivative, TCHCQ, are illustrated in Scheme 1 and Scheme 2, respectively. The new yellow compound can be synthesized in four steps. The synthesized final compound was purified by means of reprecipitation, and its structural formula was analyzed using nuclear magnetic resonance (NMR) spectroscopy and high-resolution mass (HR-MS) spectroscopy. The synthetic information for the compound is provided in Figures S1–S6. The newly developed material TCHCQ, intended for commercialization as an image sensor color filter, was researched with the goal of synthesis efficiency. In the synthetic process, we refrained from using column chromatography and recrystallization purification methods. Instead, purification was achieved through the reprecipitation method, hot filter refinement, and washing processes.

**Figure 1:**
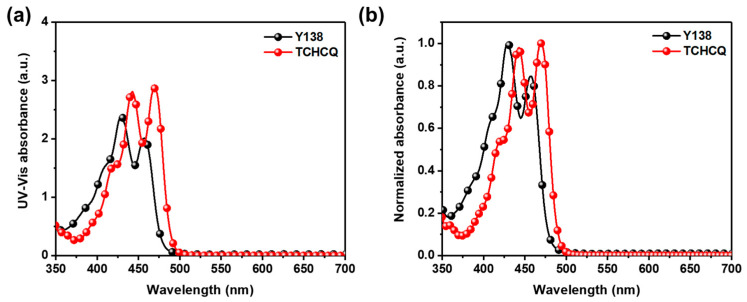
(**a**) UV-Visible absorption and (**b**) normalized UV-Visible absorption spectra of Y138 and TCHCQ in PGMEA solution (at 1.0 × 10^−5^ M).

To be used as a colorant for image sensor color filters, the newly synthesized material TCHCQ was evaluated for its solubility in the industrial solvent propylene glycol monomethyl ether acetate (PGMEA), as a good solubility in PGMEA is crucial. In this study, the PGMEA used exhibits high solubility and volatility. It is utilized in the fabrication of color resists, effectively dispersing various components such as binders, initiators, and color filters. Additionally, its evaporation after coating enables the creation of a uniform film. PGMEA is a widely used and representative solvent in the manufacturing processes of image sensors and displays. The solubility of the colorant in PGMEA solvent is determined by adding the colorant to PGMEA, stirring the mixture at room temperature for 30 min to create an oversaturated solution, filtering the solid through a filter, drying the filtered solid at 65 °C, and measuring its weight. The solubility value is then calculated by subtracting the weight of the undissolved colorant from the total weight of the injected colorant, based on the quantities of the dissolved solute and PGMEA solvent. The new yellow colorant TCHCQ showed an improved solubility of 0.165 M, 1.14 times higher than that of Y138, confirming good solubility, while the solubility of the commercial material Y138 in PGMEA was 0.144 M. Colorants used in the image sensor industry typically require a molar extinction coefficient value (ε_max_) of at least 1.0 × 10^4^ L/mol·cm. The UV-Vis absorption data in the solution state, illustrating ε_max_ characteristics, are summarized in Figure 1 and Figure S7 and Table 1. Y138, a well-known commercially available material among quinophthalone derivatives, has been widely used as a yellow color filter additive. The molar extinction coefficient for Y138 was determined to be 2.37 × 10^5^ L/mol·cm at its peak absorption wavelength of 457 nm, indicating excellent performance. The synthesized material TCHCQ also exhibited a molar extinction coefficient value (ε_max_) of 2.87 × 10^5^ L/mol·cm at the maximum absorption wavelength of 470 nm. Consequently, the newly synthesized colorant TCHCQ not only satisfied the concentration of 1 × 10^−5^ M in PGMEA but also demonstrated a molar extinction coefficient value exceeding 1.0 × 10^4^ L/mol·cm. When compared to Y138, TCHCQ showed a 1.21-fold increase in the molar extinction coefficient value. To minimize color interference between adjacent pixels due to the demand for finer image sensor pixels, it is essential to maintain high color purity. For yellow colorants, in addition to achieving high absorption at 435 nm, as mentioned earlier, another criterion for assessing high color purity is observing the absorption intensity slope between the maximum absorption peak around 500 nm and the wavelength where absorption does not occur near 500 nm. The values for the wavelengths where absorption does not occur near 500 nm for both Y138 and TCHCQ, shown as the absorption band edge in Figure 1b, were 479 nm and 490 nm, respectively. Comparing TCHCQ to Y138, it was observed that the absorption intensity slope of TCHCQ is steeper than that of Y138, indicating excellent optical characteristics. This is expected to contribute to preventing color interference between pixels and achieving high color purity. The 11 nm red-shift in the absorption band edge is interpreted as the result of a decrease in the electron-withdrawing chloride groups and an increase in the conjugation length due to the added naphthyl group. The photoluminescence (PL) of the newly synthesized compound TCHCQ in solution was examined, exhibiting a maximum PL wavelength at 517 nm. (Figure S8).

**Figure 2:**
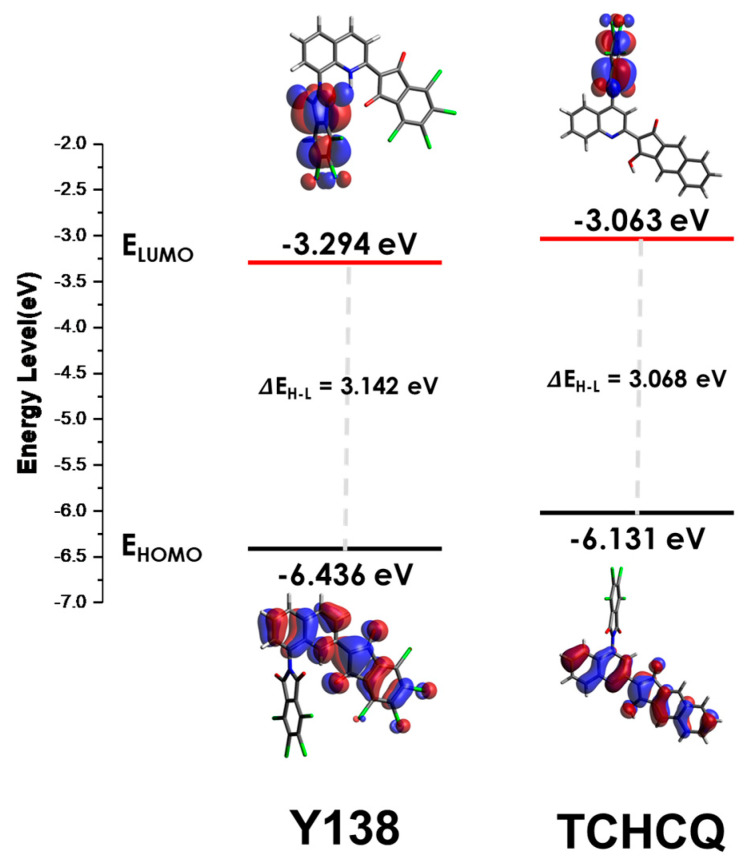
Pictorial presentation of the frontier orbitals and a plot of HOMO and LUMO energy levels for Y138 and TCHCQ (calculated at the B3LYP/6−31+G(d,p) level of theory).

The optimized structure and electronic structural characteristics of the newly synthesized yellow material can also be confirmed through molecular calculations (Figure 2 and Figure S9). The structure of Y138 exhibits a large dihedral angle of approximately 60.9° between tetrachlorophthalimide and quinoline, whereas it is interpreted to have a dihedral angle of about 0.2° between tetrachloroindenedione and quinoline, indicating their presence in the same plane. In the case of TCHCQ’s structure, it shows an increased twisted angle characteristic with a large dihedral angle of approximately 77.2° between tetrachlorophthalimide and quinoline, and an additional dihedral angle of about 17.5° between 3-hydroxy-1*H*-benz[f]inden-1-one and quinoline (Figure S9). This suggests that intermolecular stacking can be effectively prevented, inhibiting molecular packing during film formation and reducing particle aggregation. As a result, the optical properties related to color quality are expected to improve by restricting light scattering. The electron density distribution in the highest occupied molecular orbital (HOMO) of both materials is observed to be located on tetrachloroindenedione, 3-hydroxy-1*H*-benz[f]inden-1-one, and quinoline, while the lowest unoccupied molecular orbital (LUMO) has electron density located on tetrachlorophthalimide. This distribution trend is similar to that of Y138. However, the HOMO energy level for Y138 is −6.436 eV, whereas for TCHCQ, it is −6.131 eV, showing a relatively higher HOMO level by 0.305 eV in TCHCQ. This is anticipated due to a decrease in the number of Cl with a withdrawing effect compared to Y138, resulting in a higher HOMO level for TCHCQ. The bandgap of TCHCQ is measured at 3.068 eV, which is smaller than the 3.142 eV bandgap observed for Y138. This aligns with the previously mentioned red-shifted absorption range of TCHCQ compared to Y138. The redox properties of the compound were evaluated through cyclic voltammetry (CV) measurements performed using a diluted dichloromethane solution of the compound, as depicted in Figure S10. Tetrabutylammonium perchloride was used as the electrolyte, and ferrocene served as the standard substance. The HOMO energy of the compound was measured from the first oxidation potential, while the LUMO energy was obtained by subtracting the optical band gap from the HOMO value. The HOMO energies for Y138 and TCHCQ were −4.9 eV and −4.85 eV, and their LUMO energies were −2.32 eV and −2.31 eV, respectively. These values exhibit a consistent trend with the results obtained from DFT calculations.

**Figure 3:**
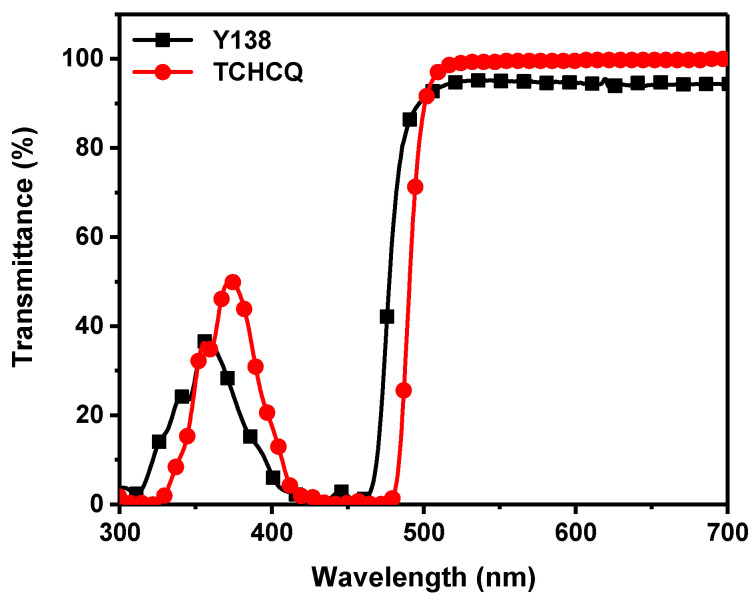
The transmittance spectra of Y138 and TCHCQ at a concentration of 1 × 10^−5^ M in PGMEA.

The transmittance data for the synthesized yellow colorant are summarized in Figure 3 and Table 2. The commercial requirements for applying yellow colorants to color filters include a transmittance of less than 5% at 435 nm in the blue region and over 90% at 530 nm in the green region. To apply a typical yellow material to a color filter, high absorption in the 400~500 nm range is necessary. Y138 exhibited a transmittance of less than 0.71% at 435 nm and over 95% at 530 nm. For TCHCQ, the transmittance was less than 0.3% at 435 nm and over 99% at 530 nm. TCHCQ demonstrated improved transmittance characteristics in the blue region near 435 nm compared to Y138. The transmittance band-edge for the new synthetic compound TCHCQ was measured at 499 nm, showing a 9 nm red-shift compared to Y138’s value of 490 nm. This red-shift is due to the introduction of the 3-hydroxy-1*H*-benz[f]inden-1-one moiety in TCHCQ, which results in a longer conjugation length despite having the same quinophthalone backbone as Y138. As a result, TCHCQ enabled low transmittance not only in the 400~470 nm range, where Y138 exhibits low transmittance, but also in the subsequent 470~490 nm range. Furthermore, the slope of the transmittance graph in the 450~525 nm range for TCHCQ showed a steeper slope compared to Y138. A steeper slope indicates an increased absorption of light at specific wavelengths, contributing to superior color purity for the newly synthesized yellow compound TCHCQ. This confirms that TCHCQ satisfies the transmittance requirements for use as a color filter in image sensors, surpassing Y138 in color purity.

**Figure 4:**
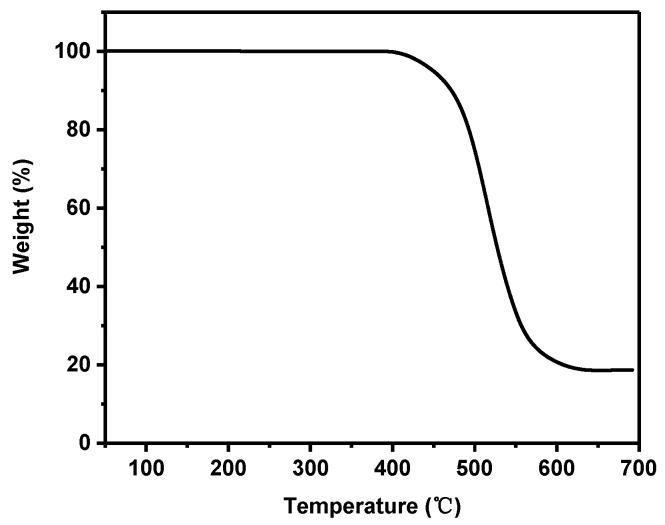
Thermogravimetric analysis (TGA) curve of TCHCQ.

To evaluate the thermal properties of the synthesized material, the decomposition temperature (T_d_) corresponding to a 5% weight loss was measured through thermogravimetric analysis (TGA). Figure 4 presents the TGA curve data. The T_d_ of the new yellow compound exhibited high thermal stability at 451 °C. The elevated thermal stability of this material ensures stability at extreme conditions, such as the required 230 °C during color filter operation and manufacturing processes. The reason for this high thermal stability lies in the utilization of the quinophthalone moiety as the main group, known for its outstanding thermal stability.

**Figure 5:**
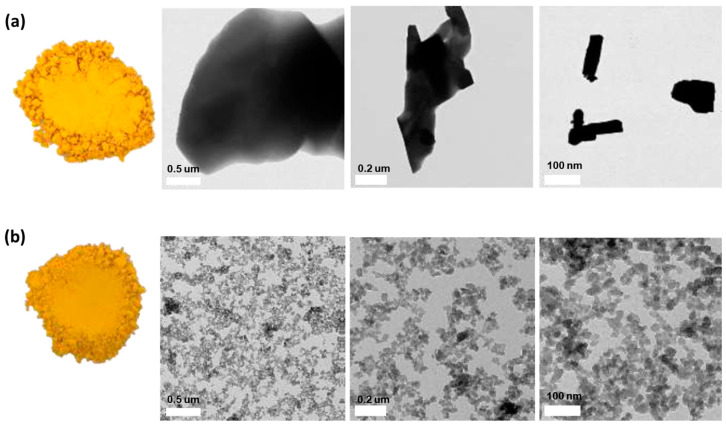
TEM images of newly synthesized material: (**a**) before the application of the pigmentation process; (**b**) after the application of the pigmentation process.

For the material to be used as a high-performance color filter, it is essential to maintain high thermal and chemical stability even when the colorant is nano-sized. To manufacture a color resist (CR) compound, a nano pigmentation process is employed after synthesizing the colorant to produce nano-sized particles. Subsequently, the CR film is manufactured by mixing the particles with a binder and photoinitiator, followed by photo-curing. The pigmentation process involves manufacturing the colorant into fine nano-sized particles, allowing for color adjustment and enhancing dispersion stability. The particle size images for the synthesized TCHCQ, applying the conventional nano-pigmentation process commonly used in general display and semiconductor applications, are depicted in Figure 5. The average particle sizes before and after the nano-pigmentation process were 200 nm and 35 nm, respectively, demonstrating a remarkable 82.5% reduction in particle size. This significant reduction in particle size is a crucial factor for achieving excellent color purity and a high absorption coefficient during CR film manufacturing, contributing to enhanced resolution and clarity when applied to color filters.

**Figure 6:**
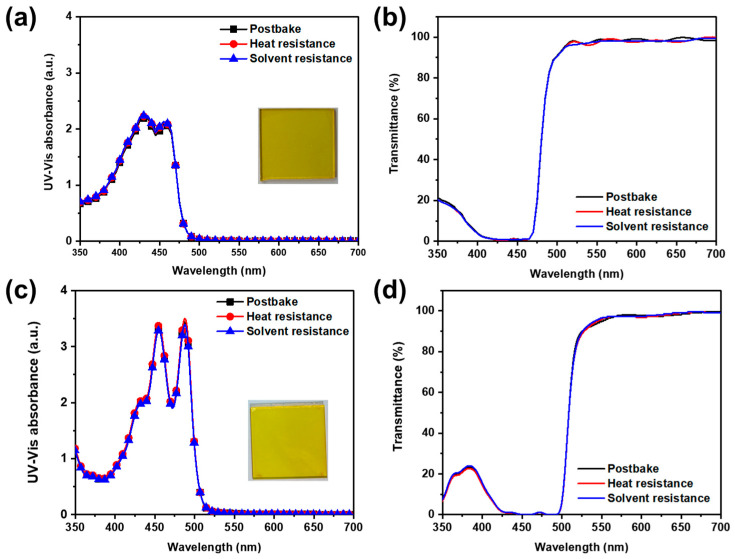
UV-Visible absorption and transmittance spectra of CR films: (**a**) and (**b**) Y138, (**c**) and (**d**) TCHCQ.

To characterize the colorant properties, CR mixtures of nano-pigmentation-processed TCHCQ and Y138 were prepared, and the performance of the resulting color filter films was evaluated. The solution was spin-coated onto a 2.5 × 2.5 cm transparent glass substrate at 1000 rpm for 10 s. After coating, a pre-bake was performed at 110 °C for 10 min, followed by exposure to UV light at 400 mJ/cm^2^ (365 nm) using a UV lamp. Subsequently, a post-bake at 220 °C for 3 min was conducted to produce the color filter. The thickness of the color resist films for Y138 and TCHCQ was 486 nm and 475 nm, respectively. To assess the thermal and chemical stability of the produced color filter, one of the characteristic evaluations, the color difference (ΔE_ab_), was measured. ΔE_ab_ indicates how well the color is maintained under conditions of thermal resistance and chemical stability during the color filter’s characteristic evaluation. In this case, a ΔE_ab_ value below 3 corresponded to excellent results, imperceptible to the human eye. The manufacturing of the color filter film and thermal stability assessment were conducted by placing the film on a metal tray. The tray was then placed in a convection oven set at 220 °C for 10 min, followed by removal and maintenance at room temperature. Subsequently, the ΔE_ab_ values before and after heat treatment were measured using the Otsuka MCPD-3000 colorimeter to confirm the thermal stability. The chemical stability was evaluated by immersing the prepared color filter film in a beaker containing PGMEA, storing it in PGMEA for 10 min, and subsequently removing and drying it. The ΔE_ab_ values before and after PGMEA solvent treatment were measured using a colorimeter, and the chemical stability was verified. The results of thermal stability and chemical stability are closely related to the progress of film curing. The characteristics of transparency, thermal resistance, and chemical resistance of the produced color filter are summarized in Figure 6 and Table 3. For the manufactured Y138 color filter, it exhibited a transmittance of less than 1% at 435 nm and over 96% at 530 nm. In the case of the color filter manufactured with TCHCQ, it showed transmittance rates of 0.97% and 91.2% at 435 nm and 530 nm, respectively. Both materials demonstrated excellent transmittance, achieving less than 1% at 435 nm and over 90% at 530 nm. The absorbance and transmittance spectra of the films for color filters using Y138 and TCHCQ showed no changes before and after the thermal stability and chemical resistance experiments. Therefore, both materials exhibited excellent thermal and chemical stability in terms of transmittance. In the thermal stability experiment, the ΔE_ab_ value for Y138 was 1.83, and in the chemical resistance experiment, it was 21.10. On the other hand, TCHCQ showed a ΔE_ab_ value of 0.60 for thermal stability and 0.52 for chemical resistance, indicating a relatively superior performance compared to Y138. While commercially available Y138 demonstrated excellent transmittance and thermal stability, it exhibited relatively lower chemical resistance. In contrast, TCHCQ exhibited excellent characteristics in both thermal stability and chemical resistance. In the thermal stability experiments, the ΔE_ab_ values for both Y138 and TCHCQ were observed to be below 3, indicating no significant changes in the maximum value and full width at half maximum of the spectra. Conversely, in terms of chemical stability, Y138 exhibited a value exceeding 3, reaching 21. This suggests a considerable reduction in the intensity of the maximum absorption wavelength. In contrast, TCHCQ showed values below 3, confirming no observable changes in the spectrum. The TCHCQ film not only showed outstanding optical properties but also demonstrated increased chemical stability, suggesting its potential for commercialization as a yellow colorant for image sensors.

## 3. Materials and Methods

### 3.1. Materials and Instrumentation

All reagents used in these experiments were purchased from Sigma-Aldrich or Tokyo Chemical Industry (TCI); their purity was 98% or higher, and they were used without further purification. Y138 was purchased from SKC Hitech and Marketing. ^1^H nuclear magnetic resonance (NMR) spectra were recorded on a Bruker Advance 300 spectrometer. ^13^C NMR data were obtained from a 600 MHz AvanceIII-600 Bruker NMR equipped with a TCI Cryoprobe. Ultraviolet–visible (UV–Vis) optical absorption spectra were recorded using a Lambda 1050 UV–Visible spectrophotometer (Perkin Elmer, Waltham, MA, USA). Thermogravimetric analysis (TGA) was performed using a TA Instruments Q5000 IR/SDT Q600 with the sample under an air atmosphere. Analysis was carried out using TRIOS v 5.0 software (TA instruments, New Castle, DE, USA). Transmission electron microscopy (TEM) images were obtained using JEM-2100F (JEOL, Tokyo, Japan). The color difference, ΔE_ab_, was measured using an Otsuka Electronics MCPD-3000 array spectrometer. The thickness of the color resist films was measured using a surface profiler (Dektak 150, Veeco, Plainview, NY, USA).

### 3.2. Computation Details

The ORCA package was utilized to perform density functional theory (DFT) calculations to carry out geometry optimization and electronic structure calculations for the molecules [17]. The applied exchange-correlation functional was the B3LYP hybrid functional, and the basis set chosen was 6−31+G(d,p).

### 3.3. Synthesis and Characterization of the Synthesized Quinophthalone Derivative

#### 3.3.1. Synthesis of 2-(2-methylquinolin-4-yl)isoindoline-1,3-dione (**1**)

In a nitrogen atmosphere, 4-aminoquinaldine (5.0 g, 31.6 mmol) was dissolved in 1,2,4-trichlorobenzene (50 mL) in a 100 mL round-bottom flask. Phthalic anhydride (4.82 g, 32.5 mmol) was added, and the resulting solution was heated to 200 °C and stirred for 12 h. After completion of the reaction, the mixture was cooled to room temperature, poured into 150 mL of hexane to precipitate the product. The resulting precipitate was filtered, washed with methanol, and vacuum-dried to obtain 6.94 g of solid (yield: 75%). ^1^H NMR (400 MHz, DMSO-d_6_): δ(ppm) = 8.04–7.99 (m, 3H), 7.96–7.92 (m, 2H), 7.85 (d, *J* = 8.4 Hz, 1H), 7.76 (ddd, *J* = 8.4, 6.9, 1.4 Hz, 1H), 7.57 (s, 1H), 7.50 (ddd, *J* = 8.2, 6.9, 1.1 Hz, 1H), 2.69 (s, 3H).

#### 3.3.2. Synthesis of 2-(2-(3-hydroxy-1-oxo-1*H*-cyclopenta[*b*]naphthalen-2-yl)quinolin-4-yl)isoindoline-1,3-dione (**2**)

In a 100 mL round-bottom flask, compound (**1**) (5 g, 0.0173 mol), naphtho [2,3-c]furan-1,3-dione (3.78 g, 0.019 mol), methyl benzoate (23.6 g, 0.173 mol), and benzoic acid (21.2 g, 0.173 mol) were added, and the mixture was stirred at 220 °C under a nitrogen atmosphere for 12 h. After the completion of the reaction, the mixture was cooled to room temperature, and methanol was added, followed by stirring for 1 h. Subsequently, the mixture was washed with methanol and acetone, filtered, and then dried in a vacuum oven to obtain 2.33 g (78% yield) of solid. ^1^H NMR (400 MHz, DMSO-d_6_): δ(ppm) = 14.43 (s, 1H), 8.77 (s, 1H), 8.26–8.12 (m, 4H), 8.09 (d, *J* = 8.3 Hz, 1H), 8.04 (dd, *J* = 5.5, 3.1 Hz, 2H), 7.95 (dd, *J* = 5.5, 3.1 Hz, 2H), 7.92 (d, *J* = 7.7 Hz, 1H), 7.89–7.85 (m, 1H), 7.62 (dd, *J* = 6.1, 3.2 Hz, 2H), 7.54–7.46 (m, 1H).

#### 3.3.3. Synthesis of 2-(4-aminoquinolin-2-yl)-3-hydroxy-1*H*-cyclopenta[*b*]naphthalen-1-one (**3**)

In a 250 mL round-bottom flask under a nitrogen atmosphere, compound (2) (2 g, 0.00426 mol), hydrazine monohydrate (4.27 g, 0.0853 mol), and 100 mL of methanol were added at room temperature. The mixture was slowly heated and stirred at 50 °C for 3 h. After the completion of the reaction, the reaction mixture was cooled to room temperature. Upon reaching room temperature, the mixture was filtered, and the solid obtained was washed with acetone. The filtered solid was then vacuum-dried at 60 °C for 12 h, yielding 1.15 g (80% yield) of solid. ^1^H NMR (400 MHz, DMSO-d_6_): δ(ppm) = 13.47 (s, 1H), 8.18 (d, *J* = 8.3 Hz, 1H), 8.05 (dd, *J* = 6.1, 3.3 Hz, 2H), 7.95 (s, 2H), 7.76–7.74 (m, 2H), 7.55 (dd, *J* = 6.2, 3.2 Hz, 2H), 7.44 (dt, *J* = 6.0, 2.3 Hz, 1H).

#### 3.3.4. Synthesis of 4,5,6,7-tetrachloro-2-(2-(3-hydroxy-1-oxo-1*H*-cyclopenta[*b*]naphthalen-2-yl)quinolin-4-yl)isoindoline-1,3-dione (TCHCQ)

In a nitrogen atmosphere, compound (**3**) (3 g, 0.0105 mol), anhydrous tetrachlorophthalic anhydride (4.97 g, 0.0147 mol), and benzoic acid (1.28 g, 0.315 mol) were added to a 100 mL round-bottom flask. The mixture was heated at 180 °C for 12 h. After the completion of the reaction, the reaction mixture was cooled to room temperature. Upon reaching room temperature, methanol was added, and the mixture was stirred for 1 h. The resulting mixture was washed with methanol and acetone, filtered, and the solid was obtained. The crude mixture was then refluxed in dimethyl sulfoxide (DMSO) solvent for 1 h and filtered. The filtered solid was washed with methanol and acetone. Additionally, the obtained solid was vacuum-dried at 60 °C for 1 day, yielding 2.54 g (40%) of a yellow solid. ^1^H NMR (400 MHz, DMSO-d_6_): δ(ppm) = 14.43 (s, 1H), 8.81 (s, 1H), 8.18 (s, 2H), 8.15 (dd, *J* = 6.1, 3.4 Hz, 2H), 8.10 (d, *J* = 8.4 Hz, 1H), 8.02 (d, *J* = 8.2 Hz, 1H), 7.89 (t, *J* = 7.8 Hz, 1H), 7.62 (dd, *J* = 6.2, 3.2 Hz, 2H), 7.55 (t, *J* = 7.7 Hz, 1H). ^13^C NMR (600 MHz, DMSO-d_6_) δ 188.87, 167.41, 166.44, 163.57, 162.38, 143.82, 138.24, 135.28, 135.20, 135.16, 130.42, 129.61, 129.57, 129.51, 129.21, 129.09, 129.05, 128.42, 127.66, 125.38, 121.44, 121.39. High-resolution mass spectrometry (HRMS) (fast atom bombardment mass spectrometry (FAB-MS), m/z): calcd. for C_30_H_12_Cl_4_N_2_O_4_, 605.95; found 606.9610 [M]^+^.

### 3.4. Fabrication and Measurement of the Colorant-Based Color Filters

To assess the thermal and chemical stability of the image sensor, colorimetric measurements were conducted under the following conditions. Nano-sized colorant dispersion was prepared using a Pascall Engineering mixer (LQFS, London, UK) with a 3 mm glass ball. A ball/colorant ratio of 100/1 was injected, and milling was performed for 48 h to achieve nano-pigmentation [18,19,20]. A color resist solution was then formulated using the nano-pigmented TCHCQ dispersion and other components. The solution for color resist preparation consisted of an acrylic binder (benzyl methacrylate/methacrylic acid, 1:1), the synthesized quinophthalone derivative (2.5 wt% of the total amount), a photoinitiator (PI OXE-57, Dongwoo Fine Chemicals, Pyeongtaek, Republic of korea, Figure S11), and the solvent (PGMEA). The solution was coated on a 2.5 cm × 2.5 cm transparent glass substrate using a MIDAS System SPIN-1200D spin-coater at 1000 rpm for 10 s. After coating, a pre-bake was performed at 110 °C for 10 min. The coated substrate was exposed to a light dose of 400 mJ/cm^2^ (at 365 nm) using a UV exposure system. Post-baking was then carried out at 220 °C for 3 min. To verify heat resistance, an additional bake was performed at 220 °C for 10 min. For chemical resistance verification, the sample was dipped in PGMEA solvent for 10 min and then dried. The ΔE_ab_ values were measured using a multi-channel spectrophotometer (Otsuka MCPD-3000).

## 4. Conclusions

The novel quinophthalone-based yellow colorant, TCHCQ, allowed for the adjustment of conjugation length by varying the number of chlorine atoms and substituting positions of tetrachloroisoindoline-1,3-dione, optimizing the performance of the yellow colorant. The synthesized TCHCQ was evaluated for optical, thermal stability, and chemical resistance properties to be applied as a colorant for image sensor color filters. TCHCQ exhibited an improved solubility in PGMEA, reaching 0.165 M, which was 1.14 times better than the commercialized Y138. Additionally, TCHCQ demonstrated excellent results, including a long-wavelength shift in the absorption band edge compared to Y138. Moreover, it showed a significantly increased molar extinction coefficient value, 1.21 times higher than that of Y138, at the maximum absorption wavelength. The thermal decomposition temperature (T_d_) of TCHCQ was 451 °C, exceeding the required 230 °C for the manufacturing process. Utilizing the nano-pigmentation process, TCHCQ particles with an average size of 35 nm were obtained, showing an outstanding 82.5% size reduction. When applied to the fabrication of color filter films through the CR solution, TCHCQ exhibited excellent optical characteristics and thermal stability similar to Y138. It exhibited improved chemical stability, indicating its potential as an excellent material for use as a yellow colorant for green color additives in image sensor color filters. Furthermore, the methods attempted in this study, involving altering the position of halogen-attached moieties and increasing the number of aromatic rings, can also lead to the optimization of absorption wavelengths when applied to other red, green, and blue colorants. Considered as a research approach capable of improving both high molar extinction coefficient values and chemical and thermal stability, these endeavors may be applied to the future synthesis of colorants for image sensors in different colors.

## Figures and Tables

**Table 1:** Optical properties of Y138 and TCHCQ.

Yellow Colorants	λ_max_ ^a^ (nm)	FWHM ^b^ (nm)	ε_max_ ^c^ (L/mol · cm)
Y138	429, 457	67	2.37 × 10^5 d^
TCHCQ	444, 470	65	2.87 × 10^5 e^

^a^ PGMEA solution (1.0 × 10^−5^ M). ^b^ The full width at half maximum. ^c^ Molar extinction coefficient at maximum absorption wavelength, ^d^ at 457 nm, and ^e^ at 470 nm.

**Table 2:** The transmittance values of Y138 and TCHCQ at 435 nm and 530 nm.

	Transmittance (%)	
	435 nm	530 nm
Y138	0.71	95.1
TCHCQ	0.30	99.3

**Table 3:** The color difference values of Y138 and TCHCQ CR films after thermal stability and chemical stability tests.

Compounds	ΔE_ab_ ^a^	ΔE_ab_ ^b^
Y138	1.83	21.10
TCHCQ	0.60	0.52

^a^ After post-baking at 220 °C for 10 min. ^b^ After immersion in PGMEA solvent for 10 min.

## Data Availability

Data are contained within the article and Supplementary Materials.

## Supplementary Materials

The following supporting information can be downloaded at: https://www.mdpi.com/article/10.3390/molecules29051100/s1, Figure S1: ^1^H NMR spectra of 2-(2-methylquinolin-4-yl)isoindoline-1,3-dione (1); Figure S2: ^1^H NMR spectra of 2-(2-(3-hydroxy-1-oxo-1*H*-cyclopenta[*b*]naphthalen-2-yl)quinolin-4-yl)isoindoline-1,3-dione (2); Figure S3: ^1^H NMR spectra of 2-(4-aminoquinolin-2-yl)-3-hydroxy-1*H*-cyclopenta[*b*]naphthalen-1-one (3); Figure S4: ^1^H NMR spectra of 4,5,6,7-tetrachloro-2-(2-(3-hydroxy-1-oxo-1*H*-cyclopenta[*b*]naphthalen-2-yl)quinolin-4-yl)isoindoline-1,3-dione (TCHCQ); Figure S5: ^13^C NMR spectra of 4,5,6,7-tetrachloro-2-(2-(3-hydroxy-1-oxo-1*H*-cyclopenta[*b*]naphthalen-2-yl)quinolin-4-yl)isoindoline-1,3-dione (TCHCQ); Figure S6: High resolution mass spectroscopy data of TCHCQ; Figure S7. The molar extinction coefficient versus wavelength graph of Y138 and TCHCQ in PGEMA solution (1.0 × 10^−5^ M); Figure S8: UV-Visible absorption and photoluminescence(PL) spectra of TCHCQ in the PGMEA solution (excitation wavelength = 470 nm); Figure S9: The optimized molecular structures calculated using B3LYP/6-31+G(d,p) of (a)Y138 and (b) TCHCQ; Figure S10: Cyclic voltammetry curve of (a) Y138 and (b) TCHCQ in dilute CH2Cl2 solutions; Figure S11: Molecular structure of photoinitiator, OXE-57.
