# Supplementary material for: Enhancement of Optical and Chemical Resistance Properties with a Novel Yellow Quinophthalone Derivative for Image Sensor Colorants
Source: Molecules. 2024 Feb 29;29(5):1100. doi: 10.3390/molecules29051100 (PMC10935299; doi:10.3390/molecules29051100)
Supplement: Supplementary file 1 [file molecules-29-01100-s001.zip › molecules-2892420-supplementary.pdf]

Supplementary Materials

Enhancement of Optical and Chemical Resistance Properties with a Novel Yellow Quinophthalone Derivative for Image Sensor Colorants

Sunwoo Park[1], Raveendra Jillella[1], Hyukmin Kwon[1], Sangwook Park[1], Hayoon Lee[1], Kiho Lee[1], and Jongwook Park[1,*]

[1]Affiliation 1; Integrated Engineering, Department of Chemical Engineering, Kyung Hee University, Gyeonggi 17104, Republic of Korea

*Correspondence: jongpark@khu.ac.kr

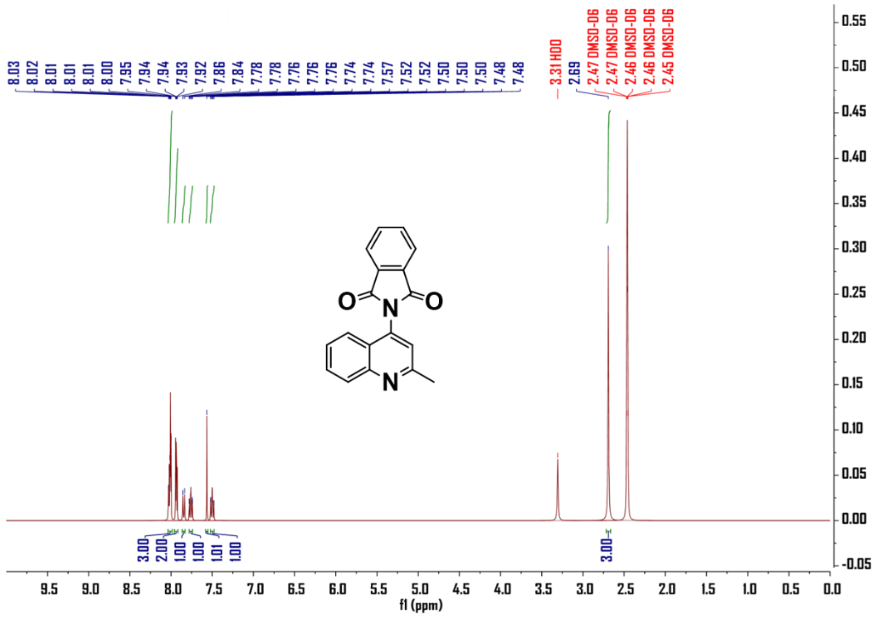

Figure S1. ^{1}H NMR spectra of 2-(2-methylquinolin-4-yl)isoindoline-1,3-dione (1)

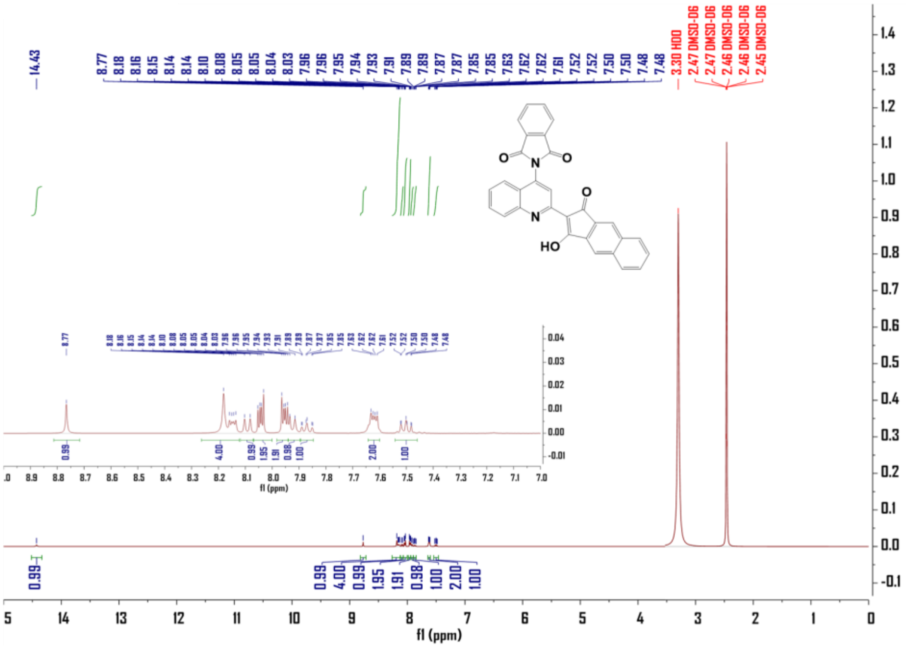

Figure S2. ^{1}H NMR spectra of 2-(2-(3-hydroxy-1-oxo-1H-cyclopenta[b]naphthalen-2-yl)quinolin-4-yl)isoindoline-1,3-dione (2)

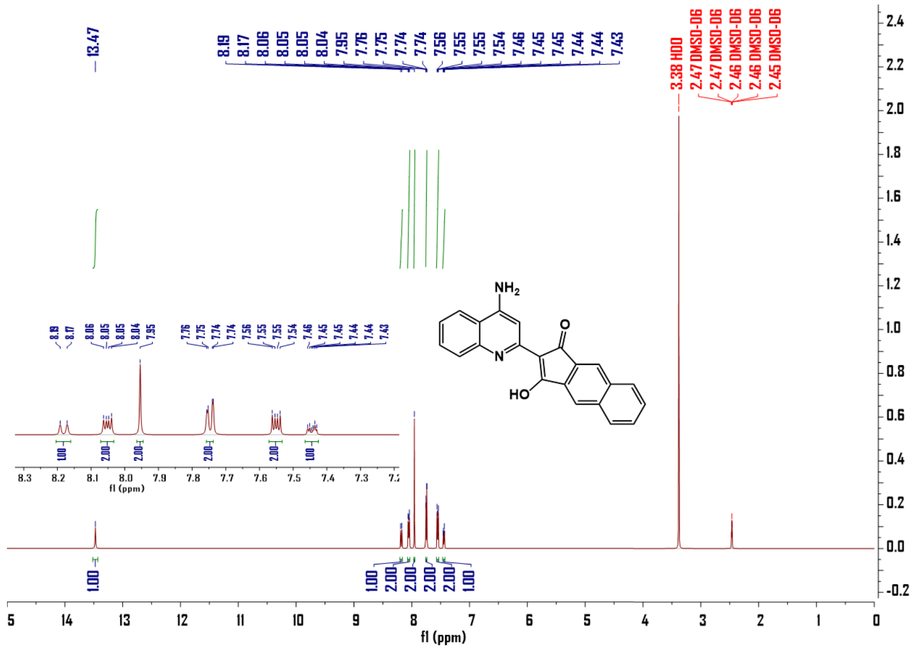

Figure S3. ^{1}H NMR spectra of 2-(4-aminoquinolin-2-yl)-3-hydroxy-1H-cyclopenta[b]naphthalen-1-one (3)

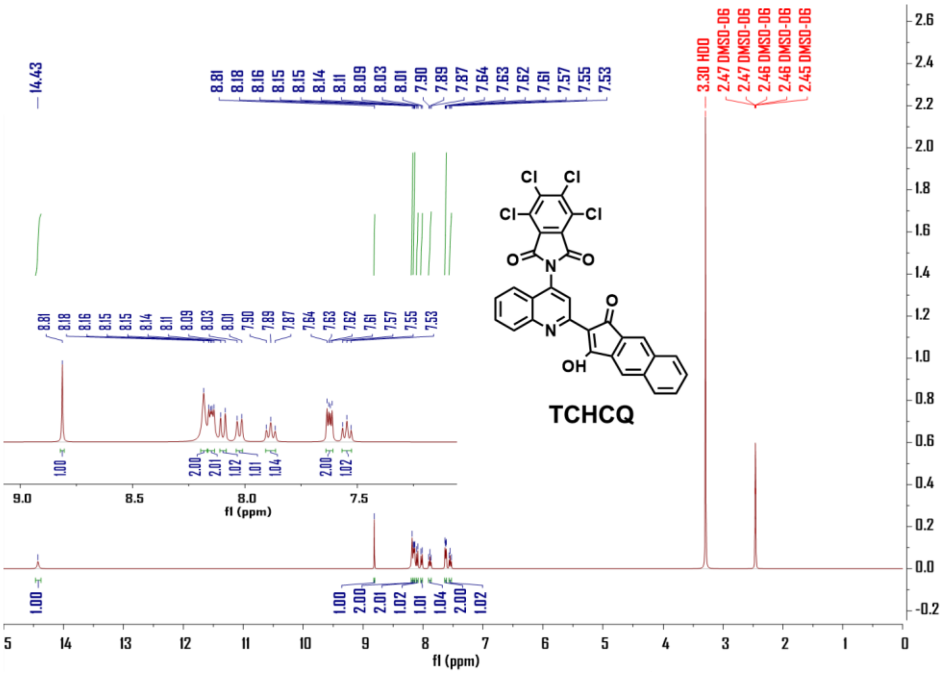

Figure S4. ^{1}H NMR spectra of 4,5,6,7-tetrachloro-2-(2-(3-hydroxy-1-oxo-1H-cyclopenta[b]naphthalen-2-yl)quinolin-4-yl)isoindoline-1,3-dione (TCHCQ)

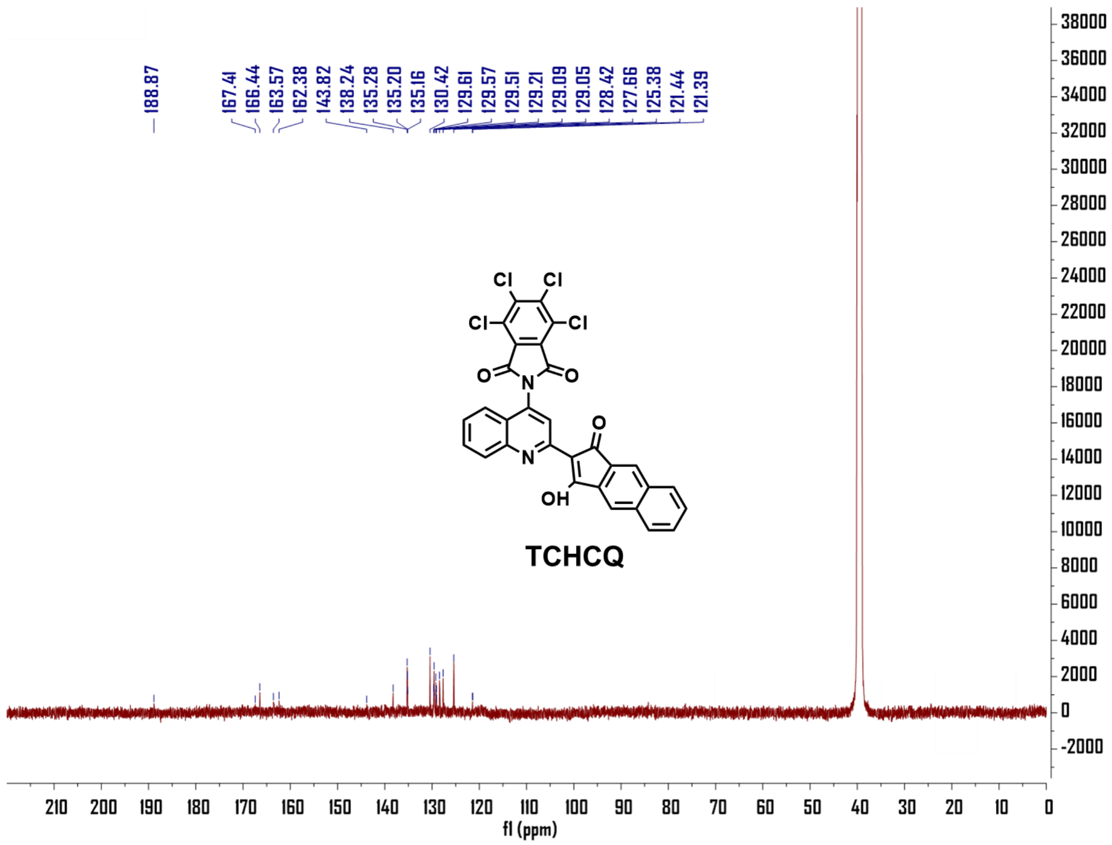

Figure S5. ^{13}C NMR spectra of 4,5,6,7-tetrachloro-2-(2-(3-hydroxy-1-oxo-1H-cyclopenta[b]naphthalen-2-yl)quinolin-4-yl)isoindoline-1,3-dione (TCHCQ).

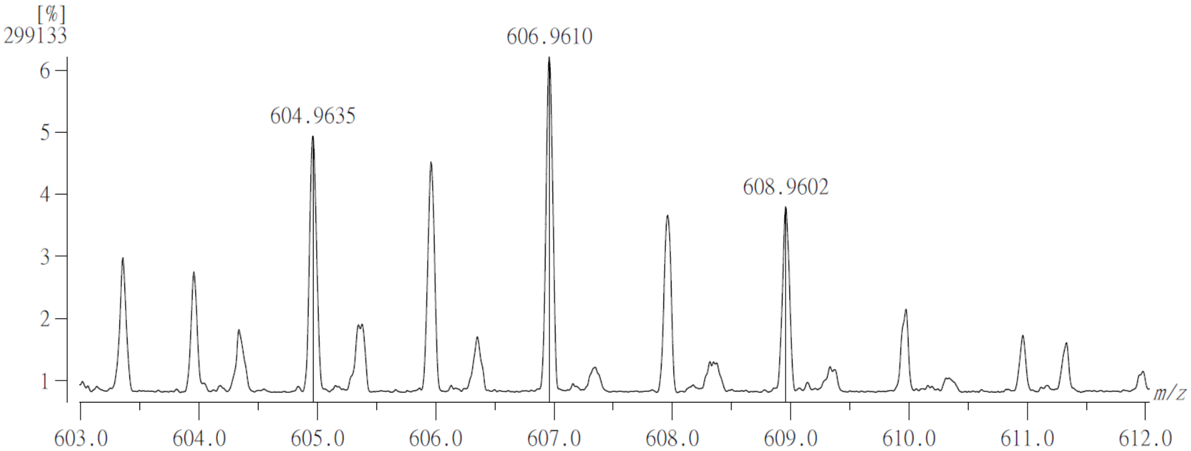

Figure S6. High resolution mass spectroscopy data of TCHCQ.

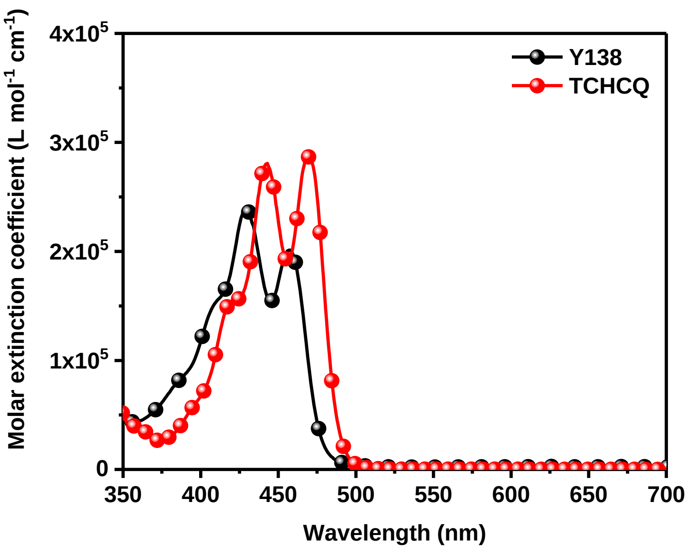

Figure S7. The molar extinction coefficient versus wavelength graph of Y138 and TCHCQ in PGEMA solution (1.0 x 10-5 M).

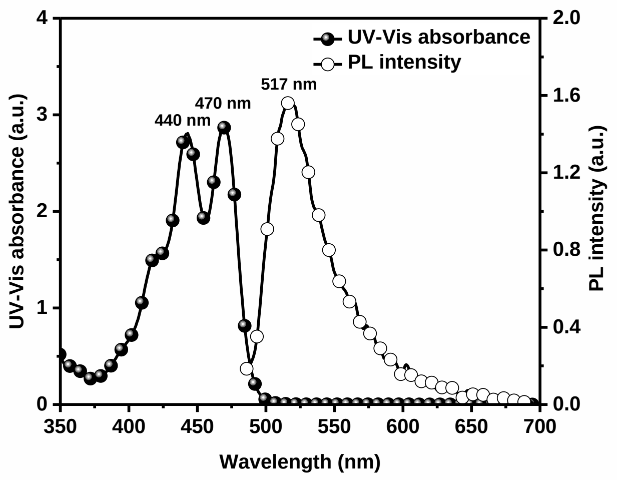

Figure S8. UV-Visible absorption and photoluminescence (PL) spectra of TCHCQ in the PGMEA solution (excitation wavelength = 470 nm).

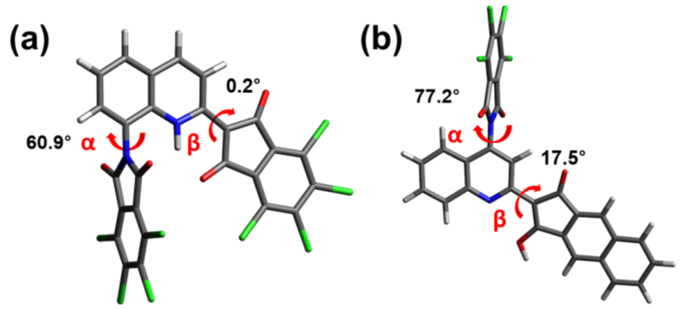

Figure S9. The optimized molecular structures calculated using B3LYP/6-31+G(d,p) of (a)Y138 and (b) TCHCQ.

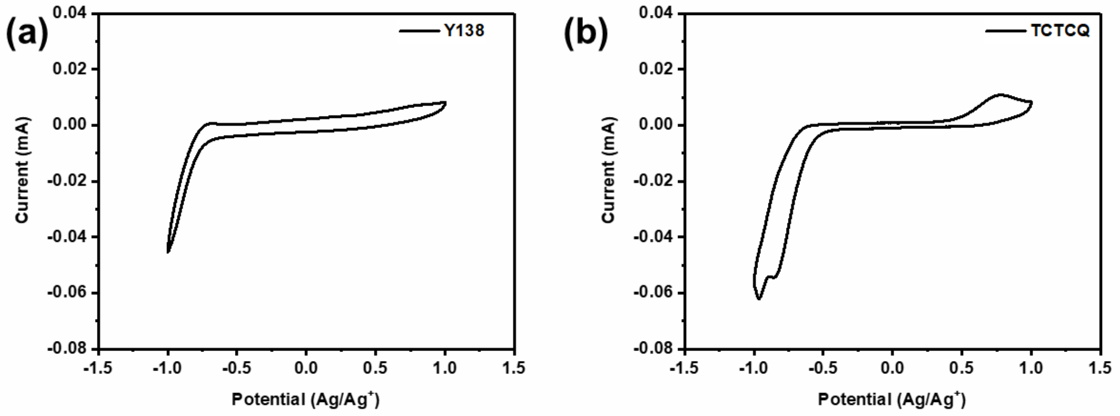

Figure S10. Cyclic voltammetry curve of (a) Y138 and (b) TCHCQ in dilute CH_2Cl_2 solutions.

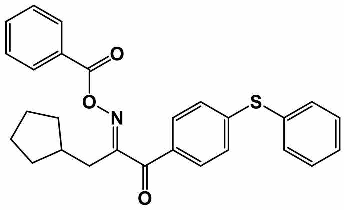

Figure S11. Molecular structure of photoinitiator, OXE-57.
